# The Admission (Neutrophil+Monocyte)/Lymphocyte Ratio Is an Independent Predictor for In-Hospital Mortality in Patients With Acute Myocardial Infarction

**DOI:** 10.3389/fcvm.2022.870176

**Published:** 2022-04-07

**Authors:** Yu Wang, Miao Yuan, Yao Ma, Congcong Shao, Yuan Wang, Mengyao Qi, Bincheng Ren, Dengfeng Gao

**Affiliations:** ^1^Department of Cardiology, The Second Affiliated Hospital, Xi’an Jiaotong University, Xi’an, China; ^2^Department of Rheumatology and Immunology, The Second Affiliated Hospital, Xi’an Jiaotong University, Xi’an, China

**Keywords:** acute myocardial infarction, (neutrophil+monocyte)/lymphocyte ratio, MIMIC-IV, mortality, prognostic indicator

## Abstract

**Purpose:**

Peripheral differential leukocyte counts are accepted prognostic indicators in patients with acute myocardial infarction (AMI). Herein, we assessed the value of the admission (neutrophil+monocyte)/lymphocyte ratio (NMLR) in predicting in-hospital mortality in these patients.

**Materials and Methods:**

Samples of patients from the Medical Information Mart for Intensive Care IV (MIMIC-IV) database meeting the criteria were included. Receiver operating characteristic (ROC) curves were plotted to explore the predictive value and the optimum cut-off value of admission NMLR. Univariate and multivariate Cox regression analyses and restricted cubic spline (RCS) were performed to determine and visualize the association between admission NMLR and in-hospital mortality. The Kaplan-Meier (KM) method was used to plot survival curves of two groups with different admission NMLR levels.

**Results:**

Samples in the non-survival group had higher admission NMLR values than samples in the survival group (12.11 [7.22–21.05] vs. 6.38 [3.96–11.25], *P* < 0.05). The area under the ROC curve (AUROC) [0.707 (95% Confidence Interval, 0.677–0.737)] was significantly better than those of other indicators related to peripheral differential leukocyte counts, and the optimal cut-off value was 8.518. Cox regression analysis identified that higher admission NMLR was an independent risk factor for in-hospital mortality. RCS visualized the uptrend and the non-linear relationship between admission NMLR and in-hospital mortality (*P*-value for non-linearity <0.05). The KM survival curve of the high admission NMLR group was significantly lower than that of the low admission NMLR group (*P* < 0.001), and the former was associated with an increased risk of in-hospital mortality compared to the latter (Hazard Ratio, 1.452; 95% Confidence Interval, 1.132–1.862; *P* < 0.05).

**Conclusion:**

An elevated admission NMLR is an independent predictor for high in-hospital mortality in patients with AMI. And it is superior to other leukocyte-related indexes.

## Introduction

Acute myocardial infarction (AMI) is a significant cause of acute mortality worldwide (1, 2). Therefore, predicting the outcome accurately is critical for clinical treatment. Regrettably, current prognostic indicators for AMI, including C-reactive protein, and brain natriuretic peptide (3), are slow and costly. Thus, it is imperative to uncover novel predictors that are faster, cheaper and easier to access, which will be beneficial to clinical practice during the course of treatment.

Inflammation triggered by the release of alarmins or damage-associated molecular patterns from dying cardiomyocytes in response to ischemic stress (4) is an accepted important factor related to mortality in AMI. Inflammatory and immune cell counts, such as peripheral total leukocyte and differential leukocyte counts, are straightforward and convenient parameters of inflammatory responses, and their prognostic value in AMI has been confirmed (5, 6).

As an emerging indicator of inflammatory and immune status, NMLR is the ratio of the sum of peripheral neutrophil and monocyte counts to peripheral lymphocyte counts. Although a few previous studies have suggested that NMLR is a prognostic indicator for diseases related to inflammatory and immune disorders (7, 8), evidence supporting the association between admission NMLR and in-hospital mortality in AMI remains scarce. Given that NMLR integrates different acute-phase inflammatory and immune responses and may comprehensively reflect the balance between damage and repair in AMI, we hypothesized that admission NMLR might represent a powerful predictor for in-hospital mortality in AMI. Given that many factors can contribute to in-hospital death, a large population is needed in multivariate analysis to take more variables into consideration. Hence, we used data from the Medical Information Mart for Intensive Care IV (MIMIC-IV) database containing numerous electronic clinical records of more than 3,80,000 patients from 2008 to 2019 (inclusive) to investigate our hypothesis. Our results finally validated the predictive value of admission NMLR in patients with AMI.

## Materials and Methods

### Data Source and Ethics Statement

We obtained data from the MIMIC-IV (Published: March 16th, 2021. Version: 1.0) database^1^ (9). As a freely available public database developed by the Massachusetts Institute of Technology Lab for Computational Physiology, MIMIC-IV integrates de-identified clinical data of patients admitted to the Beth Israel Deaconess Medical Center (BIDMC, Boston, Massachusetts, United States) from 2008 to 2019 (inclusive). The anonymous data protect patients’ privacy so well that the requirement for informed consent is waived. One of the authors completed the Collaborative Institutional Training Initiative (CITI) program course named “Data or Specimens Only Research” included in “Human Research,” passed the exam (Name ID: 9659201, Record ID: 39690061), and then obtained permission to access the dataset. We designed and conducted this study in accordance with relevant guidelines and regulations (Declaration of Helsinki).

### Samples Selection

**FIGURE 1:**
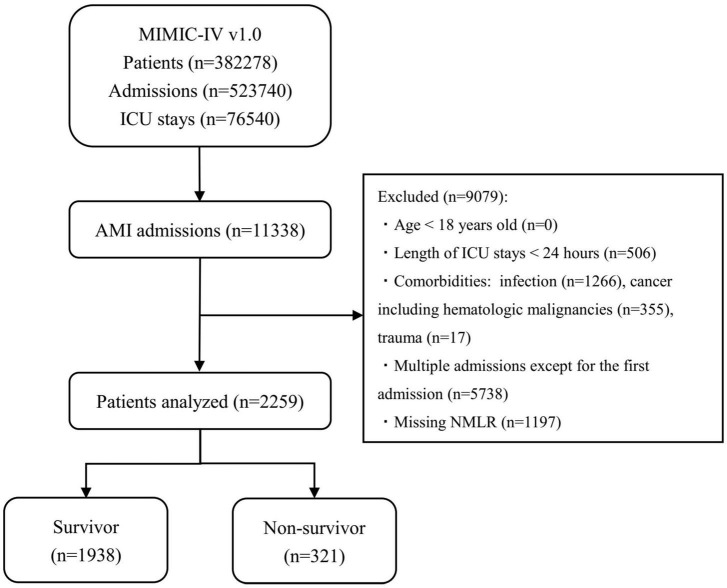
Diagram of studied samples selection steps. The MIMIC-IV (version 1.0) database includes relatively comprehensive medical information of 3,82,278 patients, 5,23,740 admissions, and 76,540 ICU stays. In total, 11,338 admissions had a discharge diagnosis of AMI. The samples of patients who met the exclusion criteria in the figure were excluded (*n* = 9,079). Eventually, 2,259 patients were included in this study, of whom 321 died. MIMIC, Medical Information Mart for Intensive Care; ICU, intensive care unit; AMI, acute myocardial infarction; NMLR, (neutrophil+monocyte)/lymphocyte ratio.

Initially, patients who had an International Classification of Diseases, 9th revision (ICD – 9) code of 410.0 or an International Classification of Diseases, 10th revision (ICD-10) code of I210.0 – I214.0 or I219.0 (the ICD code of AMI) were included. Upon further review, samples of these patients meeting the following criteria were excluded: patients who were younger than 18 years old; patients who had a secondary diagnosis of infection, cancer (including hematologic malignancies) or trauma; patients who stayed in the ICU for less than 24 h; and patients who had unobtainable documented admission NMLR. Finally, for patients admitted to the hospital more than once, only initial admission data were used. Details regarding the selection process employed in this study are given in Figure 1.

### Data Extraction

We extracted the data using structure query language (SQL) with PostgreSQL software (version 13) and Navicat Premium software (version 15). The code that supports the SQL and MIMIC-IV is publicly available.^2^ For clinical parameters with more than one result during one patient’s hospitalization, only the initial result was included. NMLR, the main factor we intended to study, was calculated as follows: NMLR = (neutrophil count+monocyte count)/lymphocyte count. Clinical parameters thought to be potential confounders were considered in this study and included demographics, comorbidities, laboratory values, vital signs, scoring systems, and treatments. The ICD codes of comorbidities and the item identification codes of laboratory values came from the official code provided by MIT Laboratory for Computational Physiology and were confirmed again by clinicians after extraction. The cardiac arrhythmia in this study included all abnormal heart rhythm types (including arrest), the ICD codes of which included ICD-9 codes of 426, 427, V1253 and ICD-10 codes of I44-I49, Z8674. Parameters with greater 30% missing values were excluded due to potential bias. Missing values, which represented less than 30% of all values considered, were replaced by the median of the variable in the subsequent analyses. The endpoint of this study was all-cause in-hospital mortality.

### Statistical Analysis

Baseline characteristics of samples were compared between survivors and non-survivors. Kolmogorov-Smirnov test was used to assess the normality of the distribution. Continuous variables are presented as the mean with standard deviation (for normal distribution) or median with interquartile range (for non-normal distribution). Student’s *t*-test or Mann-Whitney test were used as appropriate. Categorical variables are presented as counts (percentages) and were compared using the chi-square test.

Univariate and multivariate Cox regression analyses were used to identify the independent risk factors of in-hospital mortality. Variables with *P*-values less than 0.05 on univariate analysis and thought to be potential risk factors were subjected to the multivariate Cox regression model using backward Wald stepwise variable selection. Additionally, variance inflation factors (VIFs) were used to test the collinearity of variables. After the backward Wald stepwise variable selection, variables remaining constituted the multivariable-adjusted model.

Receiver operating characteristic (ROC) analysis was used to examine the association of admission NMLR with in-hospital mortality. The optimum cut-off value of admission NMLR was determined by Youden’s index. The areas under the ROC curves (AUROCs) were used to compare the admission NMLR versus other indicators related to peripheral differential leukocyte counts.

Restricted cubic spline (RCS) was used to evaluate the non-linear relationship between admission NMLR and in-hospital mortality. RCS does not need to assume linearity of the relationship between covariates and outcomes, which provides greater flexibility for fitting data (10, 11). In addition, RCS permits the model to have more complex relationships between outcomes and the variables of interest based on Cox regression while adjusting for other covariates. In this study, the RCS was based on the multivariable-adjusted Cox regression model we previously built. The spline was defined using three knots at the 5, 50, and 95th percentiles.

Samples in this study were separated into a high admission NMLR group and a low admission NMLR group according to the optimum cut-off value. Then, the Kaplan–Meier (KM) method was used to plot unadjusted survival curves of the two groups, and the log-rank test was used to compare differences between groups. The hazard ratio (HR) for in-hospital mortality was calculated using the multivariable-adjusted Cox proportional hazards model we previously built.

A *P*-value less than 0.05 was considered statistically significant, and all tests were two tailed. ROC, RCS, and KM were calculated and plotted using R software (version 4.0.4). Other statistical analyses were conducted using SPSS software (version 23).

## Results

### The Non-survival Group Had Higher Admission (Neutrophil+Monocyte)/Lymphocyte Ratio Values

Among the 2259 samples included (gender, 1496 [66.2%] male; median age, 70.71 [62.17–79.23] years; ethnicity, 1423 [63.0%] white), 321 [14.2%] died in the hospital. Baseline characteristics of samples were compared between survivors and non-survivors. In Table 1, samples in the non-survival group had higher admission NMLR values than those in the survival group (12.11 [7.22–21.05] vs. 6.38 [3.96–11.25], *P* < 0.05). In addition, many other variables also exhibited significant differences between the two groups. For example, higher neutrophil and monocyte levels were noted in the non-survival group compared with the survival group, but lymphocyte levels exhibited the opposite result.

**TABLE 1:** Baseline characteristics of the studied samples.

	All patients	Survivor	Non-survivor	*P*-value
Patients	*n* = 2259	*n* = 1938	*n* = 321	–
**Demographics**				
Age (years)	70.71 (62.17–79.23)	70.31 (62.00–78.43)	73.93 (65.35–84.04)	**<0.001**
Gender (male, n[%])	1496 [66.2%]	1302 [67.2%]	194 [60.4%]	**0.018**
Ethnicity (white, n[%])	1423 [63.0%]	1231 [63.5%]	192 [59.8%]	0.203
BMI (kg/m^2^)	28.07 (25.56–31.06)	28.07 (25.65–31.02)	28.07 (25.15–31.53)	0.977
Marital status (married, n[%])	1078 [47.7%]	951 [49.1%]	127 [39.6%]	**0.002**
Admission type (emergency, n[%])	759 [33.6%]	604 [31.2%]	155 [48.3%]	**<0.001**
**Comorbidities**				
Hypertension (n[%])	960 [42.5%]	874 [45.1%]	86 [26.8%]	**<0.001**
Cardiac arrhythmia (n[%])	1255 [55.6%]	1024 [52.8%]	231 [72.0%]	**<0.001**
Congestive heart failure (n[%])	1180 [52.3%]	980 [50.6%]	201 [62.6%]	**<0.001**
Valvular disease (n[%])	538 [23.8%]	457 [23.6%]	81 [25.2%]	0.520
Pulmonary hypertension (n[%])	105 [4.6%]	76 [3.9%]	29 [9.0%]	**<0.001**
Chronic pulmonary disease (n[%])	574 [25.4%]	471 [24.3%]	103 [32.1%]	**0.003**
Peripheral vascular disease (n[%])	354 [15.7%]	286 [14.8%]	68 [21.2%]	**0.003**
Cerebrovascular disease (n[%])	302 [13.4%]	238 [12.3%]	64 [19.9%]	**<0.001**
Diabetes mellitus (n[%])	978 [43.3%]	834 [43.0%]	144 [44.9%]	0.541
Renal disease (n[%])	653 [28.9%]	528 [27.2%]	125 [38.9%]	**<0.001**
Severe liver disease (n[%])	48 [2.1%]	25 [1.3%]	23 [7.2%]	**<0.001**
Charlson comorbidity index	7 (5–9)	6 (5–8)	8 (6–10)	**<0.001**
**Laboratory variables**				
NMLR	6.97 (4.22–12.43)	6.38(3.96–11.25)	12.11 (7.22–21.05)	**<0.001**
WBC (K/μL)	12.20 (8.90–16.40)	11.90 (8.78–15.80)	14.30 (10.60–19.65)	**<0.001**
Monocyte (K/μL)	1.17 (0.59–39.60)	1.13 (0.57–39.20)	1.52 (0.78–41.47)	**0.002**
Neutrophil (K/μL)	15.05 (8.65–785.16)	14.64 (8.40–766.82)	18.11 (10.65–939.28)	**<0.001**
Lymphocyte (K/μL)	2.49 (1.29–106.53)	2.64 (1.36–112.08)	1.74 (0.86–78.99)	**<0.001**
Platelet (K/μL)	206 (164–259)	206 (166–256)	204 (144–277)	0.305
Hematocrit (%)	35.9 (31.1–40.4)	36.3 (31.5–40.6)	33.7 (29.4–38.3)	**<0.001**
Hemoglobin (g/dL)	11.8 (10.2–13.4)	12.0 (10.3–13.5)	10.9 (9.4–12.3)	**<0.001**
MCH (pg)	30.2 (28.9–31.4)	30.2 (29.0–31.4)	30.0 (28.5–31.2)	**0.020**
MCHC (%)	33.0 (32.0–33.9)	33.0 (32.1–34.0)	32.3 (31.2–33.2)	**<0.001**
MCV (fL)	91 (88–95)	91 (88–95)	92 (88–97)	**0.001**
RBC (m/uL)	4.01 (3.39–4.51)	4.05 (3.44–4.53)	3.68 (3.14–4.23)	**<0.001**
RDW (%)	13.9 (13.2–15.0)	13.9 (13.2–14.8)	14.6 (13.7–16.5)	**<0.001**
Troponin T (ng/mL)	0.50 (0.21–1.08)	0.50 (0.22–0.98)	0.50 (0.20–1.55)	0.075
CKMB (ng/mL)	12.0 (7.0–26.0)	12.0 (6.0–24.0)	12.0 (7.0–41.0)	**0.021**
Anion Gap (mEq/L)	15 (13–18)	15 (13–17)	17 (15–21)	**<0.001**
Bicarbonate (mEq/L)	23 (20–26)	23 (21–26)	21 (18–24)	**<0.001**
BUN (mg/dL)	22 (16–35)	21 (15–31)	32 (20–53)	**<0.001**
Calcium (mg/dL)	8.6 (8.3–9.0)	8.6 (8.4–9.0)	8.3 (7.7–8.7)	**<0.001**
Chloride (mEq/L)	103 (100–106)	103 (100–106)	102 (97–106)	**0.002**
Creatinine (mg/dL)	1.1 (0.9–1.6)	1.1 (0.8–1.5)	1.6 (1.1–2.3)	**<0.001**
Glucose (mg/dL)	137 (110–187)	137 (109–178)	163 (124–233)	**<0.001**
Sodium (mEq/L)	139 (136–141)	139 (136–141)	138 (135–141)	**0.014**
Potassium (mEq/L)	4.2 (3.9–4.6)	4.2 (3.9–4.5)	4.3 (3.8–5.0)	**<0.001**
**Vital signs**				
SBP (mmHg)	118 (105–133)	118 (105–132)	118 (103–133)	0.258
DBP (mmHg)	63 (55–75)	63 (55–74)	63 (55–76)	0.623
MBP (mmHg)	81 (71–92)	81 (71–92)	80 (69–90)	0.213
Heart rate (bpm)	83 (74–95)	82 (74–94)	91 (78–108)	**<0.001**
Respiratory rate (insp/min)	18 (15–22)	17 (15–21)	21 (17–25)	**<0.001**
Temperature (°C)	36.61 (36.39–36.94)	36.61 (36.39–36.91)	36.61 (36.33–36.94)	0.649
SpO_2_ (%)	98 (95–100)	99 (96–100)	97 (94–100)	**<0.001**
**Scoring systems**				
SOFA	5 (3–8)	5 (3–8)	9 (6–13)	**<0.001**
SIRS	3 (2–3)	3 (2–3)	3 (3–4)	**<0.001**
**Treatments**				
PCI (n[%])	193 [8.5%]	162 [8.4%]	31 [9.7%]	0.441
CABG (n[%])	935 [41.4%]	908 [46.8%]	27 [8.4%]	**<0.001**
IABP (n[%])	328 [14.5%]	270 [13.9%]	58 [18.1%]	0.051
RRT (n[%])	226 [10.0%]	128 [6.6%]	98 [30.5%]	**<0.001**

*P-values less than 0.05 are indicated in bold. BMI, body mass index; WBC, white blood cell; NMLR, (neutrophil+monocyte)/lymphocyte ratio; MCH, mean corpuscular hemoglobin; MCHC, mean corpuscular hemoglobin concentration; MCV, mean corpuscular volume; RBC, red blood cell; RDW, red cell distribution width; CKMB, creatine kinase - MB isoenzyme; BUN, blood urea nitrogen; SBP, systolic blood pressure; DBP, diastolic blood pressure; MBP, mean blood pressure; SpO_2_, pulse oxygen saturation; SOFA, Sequential Organ Failure Assessment score; SIRS, Systemic Inflammatory Response Syndrome score; PCI, percutaneous coronary intervention; CABG, coronary artery bypass graft; IABP, intra-aortic balloon pump; RRT, renal replacement therapy.*

### A Higher Admission (Neutrophil+Monocyte)/Lymphocyte Ratio Value Was an Independent Risk Factor for In-Hospital Mortality

We calculated HR and confidence intervals (CIs) of variables included in this study. In Table 2, univariate Cox regression analysis showed that many variables were associated with in-hospital mortality. Subsequently, multivariate Cox regression analysis identified that higher admission NMLR (HR, 1.0082; 95% CI, 1.0026–1.0138; *P* = 0.004), older age (HR, 1.0311; 95% CI, 1.0210–1.0412; *P* < 0.001), emergency admission type (HR, 1.5111; 95% CI, 1.2015–1.9003; *P* < 0.001), chronic pulmonary disease (HR, 1.3048; 95% CI, 1.0243–1.6622; *P* = 0.031), severe liver disease (HR, 1.8495; 95% CI, 1.1695–2.9249; *P* = 0.009), higher CKMB (HR, 1.0025; 95% CI, 1.0012–1.0038; *P* < 0.001), higher anion gap (HR, 1.0248; 95% CI, 1.0028–1.0474; *P* = 0.027), higher glucose (HR, 1.0010; 95% CI, 1.0002–1.0019; *P* = 0.014), higher respiratory rate (HR, 1.0227; 95% CI, 1.0049–1.0407; *P* = 0.012), higher SOFA score (HR, 1.0799; 95% CI, 1.0488–1.1119; *P* < 0.001), IABP (HR, 1.8537; 95%CI, 1.3544–2.5371; *P* < 0.001), and RRT (HR, 1.5353; 95%CI, 1.1668–2.0201; *P* = 0.002) were independent risk factors for in-hospital mortality. In contrast, higher MCHC (HR, 0.9007; 95%CI, 0.8397–0.9661, *P* = 0.003) and CABG (HR, 0.2090; 95%CI, 0.1379–0.3168; *P* < 0.001) were identified as protective factors. The maximum VIF value was 1.365, indicating no collinearity between admission NMLR and other variables. Finally, these 14 variables associated with in-hospital mortality in multivariate Cox regression analysis constituted the multivariable-adjusted Cox regression model that would be used in subsequent studies.

**TABLE 2:** Univariate and multivariate analysis of prognostic variables.

	Univariate analysis		Multivariate analysis	
Prognostic variables	HR (95%CIs)	*P*-value	HR (95%CIs)	*P*-value
**Demographics**				
Age	1.0202 (1.0107–1.0298)	<0.001	1.0311 (1.0210–1.0412)	**<0.001**
Gender (male)	0.8262 (0.6605–1.0337)	0.095		
Ethnicity (white)	0.8842 (0.7070–1.1059)	0.281		
Marital status (married)	0.7588 (0.6066–0.9493)	0.016	0.9003 (0.7134–1.1361)	0.376
Admission type (emergency)	1.9761 (1.5869–2.4607)	<0.001	1.5111 (1.2015–1.9003)	**<0.001**
BMI	1.0115 (0.9946–1.0286)	0.183		
**Comorbidities**				
Hypertension	0.6050 (0.4719–0.7756)	<0.001	0.8510 (0.6567–1.1028)	0.222
Cardiac arrhythmia	1.6120 (1.2618–2.0594)	<0.001	1.0437 (0.8053–1.3527)	0.746
Congestive heart failure	1.1881 (0.9463–1.4916)	0.138		
Valvular disease	0.8345 (0.6481–1.074)	0.161		
Pulmonary hypertension	1.4850 (1.0128–2.1774)	0.043	1.4136 (0.9557–2.0911)	0.083
Chronic pulmonary disease	1.3267 (1.0494–1.6774)	0.018	1.3048 (1.0243–1.6622)	**0.031**
Peripheral vascular disease	1.2580 (0.9621–1.6450)	0.093		
Cerebrovascular disease	1.2066 (0.9162–1.5891)	0.181		
Diabetes mellitus	1.0161 (0.8153–1.2665)	0.887		
Renal disease	1.1924 (0.9512–1.4947)	0.127		
Severe liver disease	2.5193 (1.6443–3.8598)	<0.001	1.8495 (1.1695–2.9249)	**0.009**
Charlson comorbidity index	1.1101 (1.0655–1.1565)	<0.001	0.9878 (0.9304–1.0488)	0.689
**Laboratory variables**				
NMLR	1.0191 (1.0145–1.0238)	<0.001	1.0082 (1.0026–1.0138)	**0.004**
WBC	1.0107 (1.0053–1.0161)	<0.001	1.0071 (0.9980–1.0164)	0.126
Monocyte	1.0032 (1.0006–1.0058)	0.016		
Neutrophil	1.0003 (1.0002–1.0005)	<0.001		
Lymphocyte	0.9998 (0.9985–1.0011)	0.757		
Platelet	1.0002 (0.9990–1.0014)	0.742		
Hematocrit	0.9861 (0.9703–1.0021)	0.088		
Hemoglobin	0.9287 (0.8857–0.9736)	0.002		
MCH	0.9786 (0.9373–1.0217)	0.325		
MCHC	0.8184 (0.7630–0.8778)	<0.001	0.9007 (0.8397–0.9661)	**0.003**
MCV	1.0209 (1.0041–1.0380)	0.015		
RBC	0.8358 (0.7305–0.9563)	0.009	0.9701 (0.8310–1.1325)	0.701
RDW	1.0867 (1.0478–1.1271)	<0.001	0.9925 (0.9463–1.0411)	0.759
Troponin T	1.0582 (1.0309–1.0861)	<0.001	1.0141 (0.9788–1.0507)	0.439
CKMB	1.0026 (1.0015–1.0037)	<0.001	1.0025 (1.0012–1.0038)	**<0.001**
Anion Gap	1.0913 (1.0723–1.1107)	<0.001	1.0248 (1.0028–1.0474)	**0.027**
Bicarbonate	0.9185 (0.8982–0.9393)	<0.001	1.0188 (0.9903–1.0480)	0.200
BUN	1.0126 (1.0090–1.0161)	<0.001	1.0009 (0.9950–1.0069)	0.763
Calcium	0.6404 (0.5633–0.7280)	<0.001	0.8890 (0.7725–1.0230)	0.100
Chloride	0.9821 (0.9642–1.0003)	0.054		
Creatinine	1.0963 (1.0497–1.1450)	<0.001	0.9557 (0.8882–1.0282)	0.225
Glucose	1.0022 (1.0015–1.0029)	<0.001	1.0010 (1.0002–1.0019)	**0.014**
Sodium	0.9852 (0.9634–1.0074)	0.190		
Potassium	1.3402 (1.1658–1.5405)	<0.001	1.0826 (0.9390–1.2482)	0.274
**Vital signs**				
SBP	0.9988 (0.9939–1.0038)	0.648		
DBP	1.0033 (0.9969–1.0097)	0.313		
MBP	0.9983 (0.9923–1.0044)	0.588		
Heart rate	1.0139 (1.0084–1.0194)	<0.001	1.0052 (0.9994–1.0110)	0.081
Respiratory rate	1.0530 (1.0377–1.0685)	<0.001	1.0227 (1.0049–1.0407)	**0.012**
Temperature	0.8796 (0.7821–0.9893)	0.032	0.9295 (0.8391–1.0296)	0.161
SpO_2_	0.9550 (0.9374–0.9729)	<0.001	1.0036 (0.9813–1.0264)	0.755
**Scoring systems**				
SOFA	1.1494 (1.1213–1.1783)	<0.001	1.0799 (1.0488–1.1119)	**<0.001**
SIRS	1.3286 (1.1673–1.5121)	<0.001	1.0424 (0.8964–1.2122)	0.590
**Treatments**				
PCI	1.1405 (0.7873–1.6521)	0.487		
CABG	0.1261 (0.0850–0.1873)	<0.001	0.2090 (0.1379–0.3168)	**<0.001**
IABP	1.3426 (1.0102–1.7843)	0.042	1.8537 (1.3544–2.5371)	**<0.001**
RRT	2.2502 (1.7582–2.8800)	<0.001	1.5353 (1.1668–2.0201)	**0.002**

*P-values less than 0.05 are indicated in bold. BMI, body mass index; WBC, white blood cell; NMLR, (neutrophil+monocyte)/lymphocyte ratio; MCH, mean corpuscular hemoglobin; MCHC, mean corpuscular hemoglobin concentration; MCV, mean corpuscular volume; RBC, red blood cell; RDW, red cell distribution width; CKMB, creatine kinase – MB isoenzyme; BUN, blood urea nitrogen; SBP, systolic blood pressure; DBP, diastolic blood pressure; MBP, mean blood pressure; SpO_2_, pulse oxygen saturation; SOFA, Sequential Organ Failure Assessment score; SIRS, Systemic Inflammatory Response Syndrome score; PCI, percutaneous coronary intervention; CABG, coronary artery bypass graft; IABP, intra-aortic balloon pump; RRT, renal replacement therapy.*

### Admission (Neutrophil+Monocyte)/Lymphocyte Ratio Was a Predictor for In-Hospital Mortality

**FIGURE 2:**
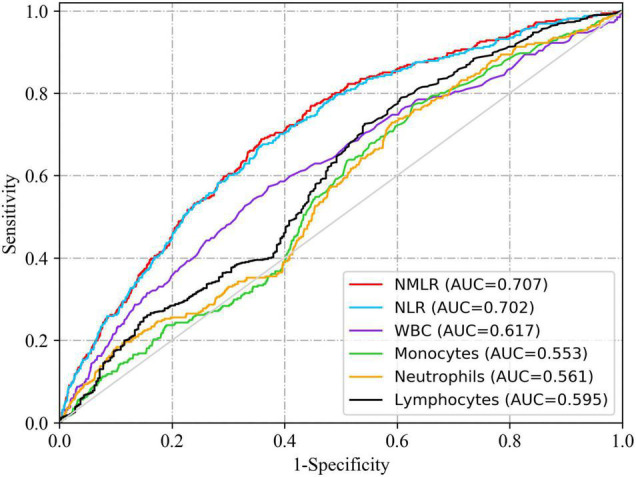
ROC curves of indicators related to peripheral differential leukocyte counts for predicting in-hospital mortality. The solid red line indicates the ROC curve of admission NMLR. The AUCs of admission NMLR and NLR were better than those of others. The follow-up DeLong’s test revealed that the AUC of admission NMLR was significantly better than that of admission NLR [0.707 (95%CI: 0.677–0.737) vs. 0.702 (95%CI: 0.672–0.732), *P* < 0.05]. NMLR, (neutrophil+monocyte)/lymphocyte ratio; AUC, area under the curve; CI, confidence interval; ROC, receiver operator characteristic.

In Figure 2, we charted ROC curves of indicators related to peripheral differential leukocyte counts for predicting in-hospital mortality. Obviously, the AUCs of admission NMLR and neutrophil to lymphocyte ratio (NLR) were better than those of others. The follow-up DeLong’s test revealed that the AUC of admission NMLR was significantly better than that of admission NLR [0.707 (95%CI: 0.677–0.737) vs. 0.702 (95%CI: 0.672–0.732), *P* < 0.05]. Accordingly, admission NMLR had considerable predictive value for in-hospital mortality. The optimum cut-off value was 8.518, with a sensitivity of 68.8% and a specificity of 64.1% (Youden’s index was maximum) (Table 3).

**TABLE 3:** Information of ROC curves in Figure 2.

Variables	AUC	95%CI	Cut-off value	Sensitivity	Specificity	Youden’s index
NMLR	0.707	0.677–0.737	8.518	0.688	0.641	0.330
NLR	0.702	0.672–0.732	8.130	0.670	0.646	0.316
WBC	0.617	0.582–0.651	13.450	0.573	0.628	0.202
Monocytes	0.553	0.521–0.585	1.100	0.626	0.492	0.118
Neutrophils	0.561	0.528–0.594	15.490	0.564	0.521	0.085
Lymphocytes	0.595	0.559–0.630	1.770	0.644	0.508	0.152

*NMLR, (neutrophil+monocyte)/lymphocyte ratio; NLR, neutrophil to lymphocyte ratio; WBC, white blood cell.*

### Higher Admission (Neutrophil+Monocyte)/Lymphocyte Ratio Levels Predicted Higher In-Hospital Mortality

**FIGURE 3:**
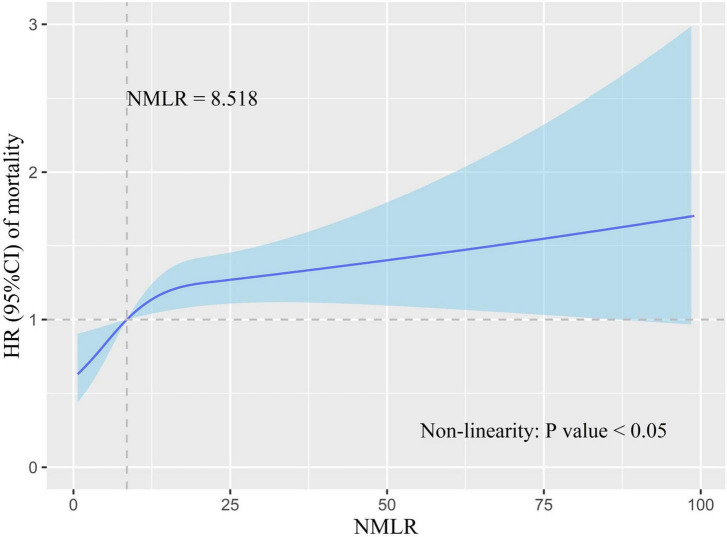
Association of admission NMLR with in-hospital mortality risk. HR is indicated by the solid blue line, and 95% CI is denoted by the shaded blue area. The model was adjusted for cofounders with a *P*-value <0.05 in multivariate Cox analysis. The reference point (HR = 1) was the point where the admission NMLR value was 8.518. The *P*-value for non-linearity was less than 0.05. HR increased with the increase in admission NMLR. However, the growth rate of HR slowed down after the admission NMLR exceeded 8.518. NMLR, (neutrophil+monocyte)/lymphocyte ratio; HR, hazard ratio; CI, confidence interval.

We used RCS to model and visualize the relation of admission NMLR with in-hospital mortality. The model was adjusted for cofounders with a *P*-value <0.05 in multivariate analysis. The reference point was an admission NMLR value of 8.518. In Figure 3, HR increased rapidly until approximately 8.518 of admission NMLR and then started to reduce the growth rate (*P*-value for non-linearity <0.05). In essence, the HR curve exhibited an upward tendency, indicating that the risk of in-hospital mortality increased with the increase in admission NMLR.

**FIGURE 4:**
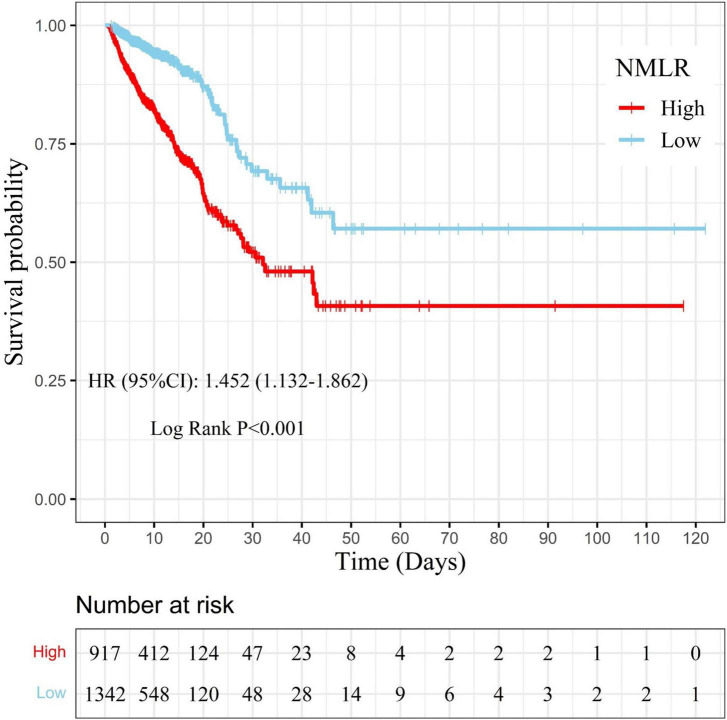
Kaplan–Meier curve for samples with different admission NMLR levels. The survival probability of samples in the high admission NMLR group (NMLR ≥8.518) is indicated by the solid red line, whereas that of samples in the low admission NMLR group (NMLR <8.518) is indicated by the solid blue line. Censors are indicated by “+”. The survival curve in the high admission NMLR group was significantly lower than that in the low admission NMLR group (log-rank test: *P* < 0.001). After adjusting for cofounders with a *P*-value <0.05 in multivariate analysis, the Cox regression analysis showed that the high admission NMLR group was associated with an increased risk of in-hospital mortality compared to the low admission NMLR group (HR, 1.452; 95%CI, 1.132–1.862; *P* = 0.003). NMLR, (neutrophil+monocyte)/lymphocyte ratio; HR, hazard ratio; CI, confidence interval.

Samples were separated into a high admission NMLR group (NMLR ≥8.518, *n* = 917) and a low admission NMLR group (NMLR <8.518, *n* = 1342) according to the optimum cut-off value. We generated KM curves for the different groups. In Figure 4, the survival curve in the high admission NMLR group was significantly lower than that in the low admission NMLR group, and the difference between the two curves was verified by the log-rank test (*P* < 0.001). The multivariate Cox regression based on the model we built showed that the high admission NMLR group was associated with an increased risk of in-hospital mortality compared to the low admission NMLR group (HR = 1.452, 95%CI: 1.132–1.862; *P* = 0.003). Overall, a higher admission NMLR level predicted higher in-hospital mortality.

## Discussion

(Neutrophil+Monocyte)/lymphocyte ratio, which is calculated as (neutrophil+monocyte)/lymphocyte, is proposed as an emerging indicator of the inflammatory and immune status and has been proposed to predict the outcome of some diseases related to inflammatory and immune disorders in recent years. For instance, in newly diagnosed multiple myeloma patients who received bortezomib+cyclophosphamide+dexamethasone regimen therapy, Pang et al. (7) found that the high NMLR group experienced a lower median progression-free survival than the low NMLR group. In addition, in very old patients (>80 years old) with AMI, Yan et al. (8) suggested that NMLR appeared to be an independent marker for long-term cardiovascular mortality.

Similar to malignant diseases (12), there is increasing recognition that AMI is associated with inflammatory and immune responses (13–17). In the acute phase of AMI, the total peripheral leukocyte count exhibits a transient increase, and simultaneous responses of peripheral differential leukocyte counts include an increase in neutrophil and monocyte counts and a reduction in lymphocyte counts (17–19). As the predominant early responder cells, neutrophils aggregating around the infarcted tissue play a role in phagocytosing necrotic cells, which further augments the inflammatory response by enhancing the local production of inflammatory cytokines and activating inflammatory signaling pathways (20–22). In addition, neutrophils recruit circulating monocytes into the injured site and provoke changes in the phenotypes of monocytes, which can facilitate the transition from the innate immune response to the repair response (19). In experimental animal models, both T and B lymphocytes infiltrate into the damaged myocardium following AMI (23, 24) and may contribute to the reparative phase (25, 26). Nevertheless, unlike the increase in lymphocytes inside the infarction zone, a reduction in circulating lymphocytes has been reported several studies (18, 27).

Based on these theories and results, clinicians have started to focus on the clinical predictive value of circulating inflammatory and immune cells. There is abundant evidence that baseline peripheral total and differential leukocyte counts are associated with mortality in patients with AMI (5, 6, 28, 29). Dragu et al. (30) found that elevated baseline total leukocyte, neutrophil and monocyte counts and reduced baseline lymphocyte counts portended high mortality risks. Azab et al. (5) suggested that the neutrophil to lymphocyte ratio was the strongest predictor of long-term mortality in patients with non-ST-segment elevation myocardial infarction. In another well-conducted study, Pasquale et al. (31) investigated the relationship between hyperglycemia, circulating inflammatory cells related indexes, and infarct size in a cohort of AMI patients that included myocardial infarction with non-obstructive coronary arteries (MINOCA). They found that similar increased values of inflammatory parameters were detected at admission in hyperglycemic patients (both obstructive AMI and MINOCA), while the levels of inflammatory parameters decreased among the MINOCA cohort after 24h. And the hyperglycemic MINOCA cases exhibited modest myocardial damage. It seems that an early decline in inflammation level indicates a better outcome, and the persistent inflammatory burden indicates a poor outcome. Since monocytes are often considered chronic inflammatory cells, we supposed that the inclusion of monocyte count in NMLR might indicate both the acute inflammatory status and the persistent inflammatory burden.

These observations inspired us to verify the ability of admission NMLR to predict in-hospital mortality in AMI. 55 variables were included in this study. After being analyzed by the Cox regression method, gender, ethnicity, marital status, BMI and some other features were excluded. The predictive value of these excluded variables may depend on other variables. Ultimately, our results validated the predictive value of admission NMLR. First, baseline characteristics analysis showed that the non-survival group had higher admission NMLR values than the survival group. When analyzed separately, neutrophil and monocyte levels in the non-survival group were higher than those in the survival group, but lymphocytes exhibited the opposite result. Then, Cox regression analyses revealed that a higher admission NMLR value was an independent risk factor for in-hospital mortality. Furthermore, ROC curves revealed that the AUC of admission NMLR was significantly better than those of NLR, WBC, neutrophils, monocytes and lymphocytes. The AUC of admission NMLR was greater than 0.7, indicating that admission NMLR has considerable predictive value for in-hospital mortality. Finally, the RCS model and KM curves of samples with different admission NMLR groups demonstrated that a higher admission NMLR level predicted higher in-hospital mortality.

Data with MINOCA and troponin-positive with non-obstructive coronary arteries (TpNOCA) diagnosis were unavailable in the MIMIC-IV database. After manually reviewing the records of patients without any coronary treatments, coronary dissection can be seen in the diagnostic lists of some patients. These patients should be diagnosed as MINOCA. Some patients had both non-ischemic causes that may be followed by positive troponins and AMI in their discharge diagnosis lists. To further explore the influence of TpNOCA, especially those caused by non-coronary factors such as myocardial or extracardiac disorders, we deleted 104 subjects with pulmonary embolism, myocarditis, or Tako-tsubo diseases. After performing the whole analysis process again, the relationship between admission NMLR and in-hospital mortality remained broadly similar (see Supplementary Materials). The prognostic value of the admission NMLR is robust.

To the best of our knowledge, the present study is the first to report the value of admission NMLR in predicting in-hospital mortality in patients with AMI. The relatively large patient population in this study reduced the selection bias to some extent, which further strengthened the conclusion. More importantly, as a clinical parameter, the admission NMLR is fast, cheap and easy to obtain.

Although these results provide forceful evidence regarding the predictive value of admission NMLR in AMI, in this retrospective single-center study, a relatively small piece of missing data that were replaced with medians may cause a loss in practical significance. Meanwhile, despite large amounts of variates included in this study, a lack of smoking, drinking and medical therapy history due to the unavailability from the MIMIC-IV database may also affect the practical significance. Another limitation was that we did not stratify the population because of the small number of deaths. Thus, a prospective multicenter study with complete data is needed for further validation. In addition, short- and long-term mortality and other important outcomes that could not be investigated due to unavailable out-of-hospital death in the MIMIC-IV v1.0 database deserve further investigation in the future.

## Conclusion

In the current study, we found that the admission NMLR is an independent predictor for in-hospital mortality in patients with AMI, and an increased admission NMLR level is associated with high mortality. For patients with elevated NMLR at admission, adequate attention and close monitoring should be provided during the entire hospital stay.

## Data Availability Statement

Publicly available datasets were analyzed in this study. This data can be found here: We obtained data from the MIMIC-IV (Published: March 16th, 2021. Version: 1.0) public database. Researchers can obtain permission to access the dataset after they complete the Collaborative Institutional Training Initiative (CITI) program course named “Data or Specimens Only Research” included in “Human Research” and pass the exam. https://doi.org/10.13026/s6n6-xd98.

## Ethics Statement

Ethical review and approval was not required for this study on human participants in accordance with the local legislation and institutional requirements. Written informed consent for participation was not required for this study in accordance with the national legislation and the institutional requirements.

## Author Contributions

YuW, MQ, BR, and DG: conceptualization. YuW, MY, and YM: methodology analysis. YuW and MY: writing. YuanW and CS: visualization. All the authors have read and agreed to submit the manuscript.

## Conflict of Interest

The authors declare that the research was conducted in the absence of any commercial or financial relationships that could be construed as a potential conflict of interest.

## Publisher’s Note

All claims expressed in this article are solely those of the authors and do not necessarily represent those of their affiliated organizations, or those of the publisher, the editors and the reviewers. Any product that may be evaluated in this article, or claim that may be made by its manufacturer, is not guaranteed or endorsed by the publisher.

## Footnotes

1
https://doi.org/10.13026/s6n6-xd98

2
https://github.com/MIT-LCP/mimic-code
