# Supplementary material for: The Admission (Neutrophil+Monocyte)/Lymphocyte Ratio Is an Independent Predictor for In-Hospital Mortality in Patients With Acute Myocardial Infarction
Source: Front Cardiovasc Med. 2022 Apr 7;9:870176. doi: 10.3389/fcvm.2022.870176 (PMC9021423; doi:10.3389/fcvm.2022.870176)
Supplement: Supplementary file 1 [file Data_Sheet_1.pdf]

Supplementary Tables List

Supplementary Table 1: Baseline characteristics of the studied samples.

	All patients	Survivor	Non-survivor	*P* value
Patients	n=2155	n=1853	n=302	-
**Demographics**				
Age (years)	70.71 (62.20-79.36)	70.18 (61.93-78.37)	74.51 (66.71-84.38)	**< 0.001**
Gender (male, n[%])	1445 [67.05%]	1259 [67.94%]	186 [61.59%]	**0.029**
Ethnicity (white, n[%])	1354 [62.83%]	1176 [63.46%]	178 [58.94%]	0.131
BMI (kg/m^2)	28.10 (25.59-31.07)	28.10 (25.74-31.08)	28.10 (25.09-31.00)	0.637
Marital status (married, n[%])	1037 [48.12%]	917 [49.49%]	120 [39.74%]	**0.002**
Admission type (emergency, n[%])	719 [33.36%]	569 [30.71%]	150 [49.67%]	**< 0.001**
**Comorbidities**				
Hypertension (n[%])	909 [42.18%]	829 [44.74%]	80 [26.49%]	**< 0.001**
Cardiac arrhythmia (n[%])	1199 [55.64%]	979 [52.83%]	220 [72.85%]	**< 0.001**
Congestive heart failure (n[%])	1122 [52.06%]	932 [50.30%]	190 [62.91%]	**< 0.001**
Valvular disease (n[%])	515 [23.90%]	437 [23.58%]	78 [25.83%]	0.396
Pulmonary hypertension (n[%])	97 [4.50%]	71 [3.83%]	26 [8.61%]	**< 0.001**
Chronic pulmonary disease (n[%])	541 [25.10%]	445 [24.02%]	96 [31.79%]	**0.004**
Peripheral vascular disease (n[%])	338 [15.68%]	272 [14.68%]	66 [21.85%]	**0.001**
Cerebrovascular diseases (n[%])	275 [12.76%]	217 [11.71%]	58 [19.21%]	**< 0.001**
Diabetes mellitus (n[%])	940 [43.62%]	807 [43.55%]	133 [44.04%]	0.874
Renal disease (n[%])	627 [29.10%]	508 [27.42%]	119 [39.40%]	**< 0.001**
Severe liver disease (n[%])	46 [2.13%]	24 [1.30%]	22 [7.28%]	**< 0.001**
Charlson comorbidity index	7 (5-9)	6 (5-8)	8 (6-10)	**< 0.001**
**Laboratory variables**				
NMLR	6.88 (4.19-12.27)	6.30(3.93-11.04)	12.10 (7.23-21.20)	**< 0.001**
WBC (K/μL)	12.20 (8.90-16.30)	11.90 (8.70-15.75)	14.30 (10.60-19.63)	**< 0.001**
Monocyte (K/μL)	1.20 (0.60-40.00)	1.14 (0.58-39.64)	1.57 (0.79-41.74)	**0.004**
Neutrophil (K/μL)	15.09 (8.71-793.46)	14.72 (8.43-784.90)	18.12 (10.70-954.96)	**0.001**
Lymphocyte (K/μL)	2.57 (1.32-107.52)	2.69 (1.34-114.52)	1.75 (0.86-83.19)	**< 0.001**
Platelet (K/μL)	206 (164-259)	206 (167-256)	205 (148-342)	0.620
Hematocrit (K/μL)	36.0 (31.2-40.5)	36.4 (31.6-40.6)	33.7 (29.6-38.3)	**< 0.001**
Hemoglobin (K/μL)	11.9 (10.2-13.4)	12.0 (10.3-13.5)	10.9 (9.5-12.3)	**< 0.001**
MCH (pg)	30.2 (28.9-31.4)	30.2 (29.0-31.4)	30.0 (28.5-31.2)	**0.038**
MCHC (%)	33.0 (32.0-33.9)	33.0 (32.1-34.0)	32.3 (31.2-33.2)	**< 0.001**
MCV (fL)	91 (88-95)	91 (88-94)	93 (88-97)	**0.001**
RBC (m/uL)	3.99 (3.40-4.52)	4.00 (3.46-4.53)	3.69 (3.14-4.22)	**< 0.001**
RDW (%)	13.9 (13.2-15.0)	13.8 (13.2-14.7)	14.6 (13.7-16.5)	**< 0.001**
Troponin T (ng/mL)	0.93 (0.22-1.69)	0.94 (0.22-1.69)	0.90 (0.21-1.69)	0.990
CKMB (ng/mL)	13.0 (7.0-26.0)	13.0 (7.0-24.0)	13.0 (7.0-42.0)	**0.015**
Anion Gap (mEq/L)	15 (13-18)	15 (13-17)	17 (15-21)	**< 0.001**
Bicarbonate (mEq/L)	23 (20-26)	24 (21-26)	21 (18-24)	**< 0.001**
BUN (mg/dL)	22 (16-35)	21 (15-31)	32 (20-52)	**< 0.001**
Calcium (mg/dL)	8.6 (8.3-9.0)	8.6 (8.4-9.0)	8.3 (7.7-8.7)	**< 0.001**
Chloride (mEq/L)	103 (100-106)	103 (100-106)	102 (97-106)	**0.001**
Creatinine (mg/dL)	1.1 (0.9-1.6)	1.1 (0.8-1.5)	1.6 (1.1-2.3)	**< 0.001**
Glucose (mg/dL)	136 (110-187)	136 (108-178)	164 (122-229)	**< 0.001**

Sodium (mEq/L)	139 (136-141)	139 (136-141)	138 (135-141)	**0.016**
Potassium (mEq/L)	4.2 (3.9-4.5)	4.2 (3.9-4.5)	4.3 (3.8-4.9)	< **0.001**
**Vital signs**				
SBP (mmHg)	118 (105-132)	118 (105-132)	118 (104-133)	0.421
DBP (mmHg)	63 (55-74)	63 (54-74)	63 (55-75)	0.455
MBP (mmHg)	80 (71-92)	80 (71-92)	80 (69-90)	0.421
Heart rate (bpm)	83 (74-95)	82 (74-93)	91 (78-106)	< **0.001**
Respiratory rate (insp/min)	18 (15-22)	17 (15-21)	21 (17-25)	< **0.001**
Temperature (°C)	36.61 (36.39-36.94)	36.61 (36.39-36.94)	36.61 (36.28-36.89)	0.417
SpO_2 (%)	98 (95-100)	99 (96-100)	97 (94-100)	< **0.001**
**Scoring systems**				
SOFA	5 (3-8)	5 (3-8)	9 (6-13)	< **0.001**
SIRS	3 (2-3)	3 (2-3)	3 (3-4)	< **0.001**
**Treatments**				
PCI (n[%])	448 [20.8%]	392 [21.2%]	56 [18.5%]	0.300
CABG (n[%])	916 [42.5%]	890 [48.0%]	26 [8.6%]	< **0.001**
IABP (n[%])	320 [14.8%]	265 [14.3%]	55 [18.2%]	0.076
RRT (n[%])	208 [9.7%]	118 [6.4%]	90 [29.8%]	< **0.001**

BMI: body mass index; WBC: white blood cell; NMLR: (neutrophil + monocyte)/ lymphocyte ratio; MCH: mean corpuscular hemoglobin; MCHC: mean corpuscular hemoglobin concentration; MCV: mean corpuscular volume; RBC: red blood cell; RDW: red cell distribution width; CKMB: creatine kinase - MB isoenzyme; BUN: blood urea nitrogen; SBP: systolic blood pressure; DBP: diastolic blood pressure; MBP: mean blood pressure; SpO2: pulse oxygen saturation; SOFA: Sequential Organ Failure Assessment score; SIRS: Systemic Inflammatory Response Syndrome score; PCI: percutaneous coronary intervention; CABG: coronary artery bypass graft; IABP: intra-aortic balloon pump; RRT: renal replacement therapy.

Supplementary Table 2: Univariate and multivariate analysis of prognostic variables.

	Univariate analysis		Multivariate analysis	
Prognostic variables	HR (95%CIs)	*P* value	HR (95%CIs)	*P* value
**Demographics**				
Age	1.0248 (1.0146-1.0351)	< 0.001	1.0334 (1.0227-1.0442)	**<0.001**
Gender (male)	0.8210 (0.6510-1.0355)	0.096		
Ethnicity (white)	0.8594 (0.6829-1.0816)	0.197		
Marital status (married)	1.0048 (0.9863-1.0236)	0.61	0.9352 (0.7353-1.1895)	0.585
Admission type (emergency)	0.7514 (0.5965-0.9464)	0.015	1.5732 (1.2404-1.9954)	**<0.001**
BMI	2.1978 (1.7523-2.7566)	< 0.001		
**Comorbidities**				
Hypertension	0.6222 (0.4808-0.8051)	< 0.001	0.9004 (0.6874-1.1794)	0.446
Cardiac arrhythmia	1.6913 (1.3107-2.1824)	< 0.001	1.0769 (0.8241-1.4073)	0.587
Congestive heart failure	1.2086 (0.9553-1.5291)	0.114		
Valvular disease	0.8483 (0.6549-1.0988)	0.213		
Pulmonary hypertension	1.4203 (0.9488-2.1262)	0.088		
Chronic pulmonary disease	1.3186 (1.0348-1.6802)	0.025	1.1485 (0.8941-1.4752)	0.278
Peripheral vascular disease	1.2818 (0.9750-1.6852)	0.075		
Cerebrovascular disease	1.2184 (0.9135-1.6250)	0.179		
Diabetes mellitus	0.9435 (0.7515-1.1845)	0.617		
Renal disease	1.1757 (0.9314-1.4841)	0.173		
Severe liver disease	2.4523 (1.5838-3.7971)	< 0.001	1.5088 (0.9378-2.4273)	0.09
Charlson comorbidity index	1.1123 (1.0665-1.1602)	< 0.001	0.9800 (0.9237-1.0398)	0.505

**Laboratory variables**				
NMLR	1.0204 (1.0155-1.0253)	< 0.001	1.0088 (1.0029-1.0148)	**0.003**
WBC	1.0103 (1.0047-1.0159)	< 0.001	1.0072 (0.9982-1.0163)	0.114
Monocyte	1.0029(1.0003-1.0056)	0.028		
Neutrophil	1.0002 (1.0001-1.0004)	< 0.001		
Lymphocyte	0.9997 (0.9984-1.0010)	0.696		
Platelet	1.0002 (0.9990-1.0014)	0.666		
Hematocrit	0.9855 (0.9692-1.0022)	0.09		
Hemoglobin	0.9267 (0.8824-0.9732)	0.002		
MCH	0.9888 (0.9449-1.0346)	0.627		
MCHC	0.8177 (0.7607-0.8790)	< 0.001	0.8934 (0.8336-0.9574)	**0.001**
MCV	1.0264 (1.0089-1.0443)	0.003		
RBC	0.8274 (0.7181-0.9535)	0.009	1.0061 (0.8525-1.1874)	0.942
RDW	1.0869 (1.0466-1.1287)	< 0.001	0.9923 (0.9432-1.0439)	0.767
Troponin T	1.0517 (1.0230-1.0812)	< 0.001	1.0181 (0.9824-1.0551)	0.323
CKMB	1.0026 (1.0015-1.0037)	< 0.001	1.0028 (1.0015-1.0040)	**<0.001**
Anion Gap	1.0954 (1.0759-1.1152)	< 0.001	1.0139 (0.9895-1.0390)	0.265
Bicarbonate	0.9124 (0.8916-0.9337)	< 0.001	1.0135 (0.9780-1.0504)	0.459
BUN	1.0132 (1.0094-1.0169)	< 0.001	1.0018 (0.9957-1.0080)	0.548
Calcium	0.6013 (0.5283-0.6842)	< 0.001	0.8128 (0.7073-0.9340)	**0.003**
Chloride	0.9799 (0.9612-0.9990)	0.04	0.9697 (0.9519-0.9878)	**0.001**
Creatinine	1.1070 (1.0554-1.1611)	< 0.001	0.9474 (0.8785-1.0218)	0.162
Glucose	1.0026 (1.0018-1.0034)	< 0.001	1.0011 (1.0001-1.0020)	**0.026**
Sodium	0.9801 (0.9576-1.0031)	0.091		
Potassium	1.3468 (1.1634-1.5591)	< 0.001	1.0924 (0.9474-1.2598)	0.224
**Vital signs**				
SBP	0.9993 (0.9941-1.0045)	0.811		
DBP	1.0041 (0.9975-1.0109)	0.219		
MBP	0.9990 (0.9928-1.0053)	0.773		
Heart rate	1.0136 (1.0078-1.0194)	< 0.001	1.0053 (0.9990-1.0116)	0.096
Respiratory rate	1.0562 (1.0399-1.0726)	< 0.001	1.0261 (1.0070-1.0455)	**0.007**
Temperature	0.8099 (0.7111-0.9225)	0.002	0.8291 (0.7434-0.9247)	**0.001**
SpO_2	0.9554 (0.9371-0.9741)	< 0.001	1.0042 (0.9808-1.0280)	0.726
**Scoring systems**				
SOFA	1.1509 (1.1215-1.1810)	< 0.001	1.0944 (1.0621-1.1276)	**<0.001**
SIRS	1.3573 (1.1884-1.5502)	< 0.001	1.0022 (0.8578-1.1707)	0.978
**Treatments**				
PCI	1.1404 (0.8527-1.5252)	0.376		
CABG	0.1199 (0.0801-0.1795)	< 0.001	0.2213 (0.1452-0.3372)	**<0.001**
IABP	1.2937 (0.9657-1.7331)	0.084		
RRT	2.2464 (1.7377-2.9039)	< 0.001	1.4453 (1.0847-1.9258)	**0.012**

BMI: body mass index; WBC: white blood cell; NMLR: (neutrophil + monocyte)/ lymphocyte ratio; MCH: mean corpuscular hemoglobin; MCHC: mean corpuscular hemoglobin concentration; MCV: mean corpuscular volume; RBC: red blood cell; RDW: red cell distribution width; CKMB: creatine kinase - MB isoenzyme; BUN: blood urea nitrogen; SBP: systolic blood pressure; DBP: diastolic blood pressure; MBP: mean blood pressure; SpO_2: pulse oxygen saturation; SOFA: Sequential Organ Failure Assessment score; SIRS: Systemic Inflammatory Response Syndrome score; PCI: percutaneous coronary intervention; CABG: coronary artery bypass graft; IABP: intra-aortic balloon pump; RRT: renal replacement therapy.

Supplementary Table 3: Information of ROC curves in Supplementary Figure 1.

Variables	AUC	95%CI	Cut-off value	Sensitivity	Specificity	Youden's index
NMLR	0.711	0.680-0.741	8.522	0.689	0.648	0.336
NLR	0.706	0.675-0.737	8.145	0.669	0.652	0.321
WBC	0.617	0.581-0.652	13.600	0.560	0.631	0.191
Monocytes	0.552	0.519-0.585	1.100	0.629	0.486	0.115
Neutrophils	0.561	0.528-0.595	14.070	0.619	0.480	0.099
Lymphocytes	0.595	0.559-0.632	1.770	0.650	0.503	0.154

NMLR: (neutrophil + monocyte)/ lymphocyte ratio; NLR: neutrophil to lymphocyte ratio; WBC: white blood cell.

Supplementary Figures List

Supplementary Figure 1

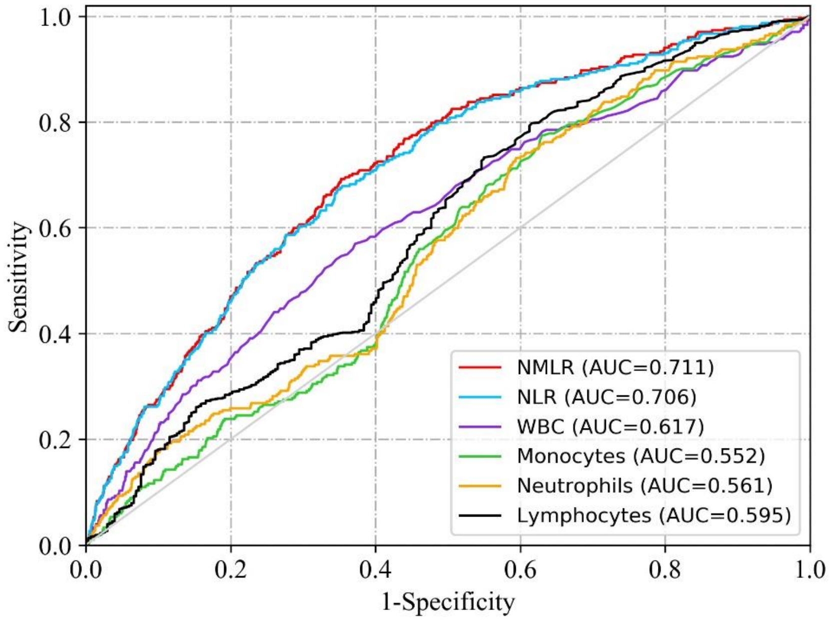

Supplementary Figure 1: ROC curves of indicators related to peripheral differential leukocyte counts for predicting in-hospital mortality.

The solid red line indicates the ROC curve of admission NMLR. The AUCs of admission NMLR and NLR were better than those of others. The follow-up DeLong's test revealed that the AUC of admission NMLR was significantly better than that of admission NLR [0.711 (95%CI: 0.680-0.741) vs. 0.706 (95%CI: 0.675-0.737), $P < 0.05$].

NMLR: (neutrophil + monocyte)/ lymphocyte ratio; AUC: area under the curve; CI: confidence interval; ROC: receiver operator characteristic.

Supplementary Figure 2

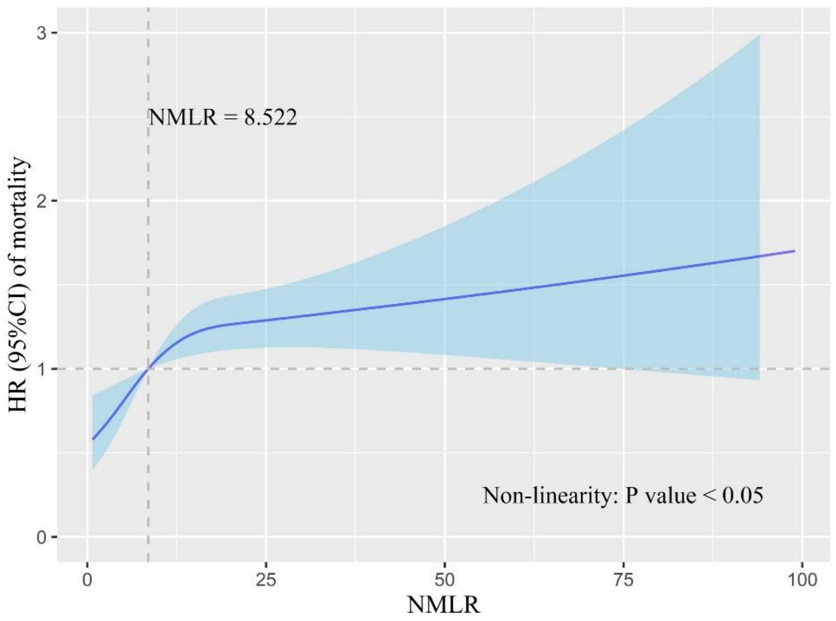

Supplementary Figure 2: Association of admission NMLR with in-hospital mortality risk.
HR is indicated by the solid blue line, and 95% CI is denoted by the shaded blue area. The model was adjusted for cofounders with a *P* value < 0.05 in multivariate Cox analysis. The reference point (HR = 1) was the point where the admission NMLR value was 8.522. The *P* value for non-linearity was less than 0.05. HR increased with the increase in admission NMLR. However, the growth rate of HR slowed down after the admission NMLR exceeded 8.522.
NMLR: (neutrophil + monocyte)/ lymphocyte ratio; HR: hazard ratio; CI: confidence interval.

Supplementary Figure 3

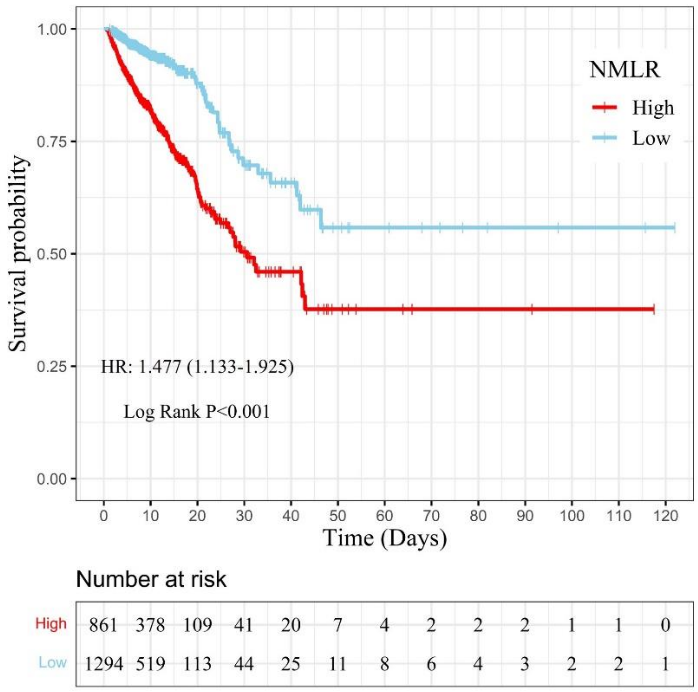

Supplementary Figure 3: Kaplan-Meier curve for samples with different admission NMLR levels.

The survival probability of samples in the high admission NMLR group (NMLR ≥ 8.522) is indicated by the solid red line, whereas that of samples in the low admission NMLR group (NMLR < 8.522) is indicated by the solid blue line. Censors are indicated by "+". The survive curve in the high admission NMLR group was significantly lower than that in the low admission NMLR group (Log-rank test: *P* < 0.001). After adjusting for cofounders with a *P* value < 0.05 in multivariate analysis, the Cox regression analysis showed that the high admission NMLR group was associated with an increased risk of in-hospital mortality compared to the low admission NMLR group (HR, 1.477; 95%CI, 1.133-1.925; *P* = 0.003).

NMLR: (neutrophil + monocyte)/ lymphocyte ratio; HR: hazard ratio; CI: confidence interval.
